# Increased systemic and peritoneal oxidative stress biomarkers in endometriosis are not related to retrograde menstruation

**DOI:** 10.1080/13510002.2019.1632603

**Published:** 2019-06-21

**Authors:** Araceli Montoya-Estrada, Cynthia F. Coria-García, Oliver P. Cruz-Orozco, Patricia Aguayo-González, Yessica D. Torres-Ramos, Héctor Flores-Herrera, Juan J. Hicks, Rafael Medina-Navarro, Alberto M. Guzmán-Grenfell

**Affiliations:** a Departamento de Inmunobioqímica, Instituto Nacional de Perinatología ‘Isidro Espinosa de los Reyes’, Ciudad de México, México; b Comisión Coordinadora de los Institutos Nacionales de Salud y Hospitales de Alta Especialidad, Ciudad de México, México; c Departamento de Metabolismo Experimental, Centro de Investigación Biomédica de Michoacán (CIBIM-IMSS), Morelia, México

**Keywords:** Oxidative damage, peritoneal fluid, retrograde menstruation, endometriosis

## Abstract

**Objetives:** The goal of this study was to determine if systemic and peritoneal oxidative stress biomarkers are related to each other and to retrograde menstruation in endometriosis.

**Methods:** Plasma and peritoneal fluid oxidative stress biomarkers and hemoglobin and erythrocytes in peritoneal fluid as retrograde menstruation indicators, were measured in 28 patients with endometriosis and 23 without endometriosis.

**Results:** In the peritoneal fluid, carbonyls and lipohydroperoxides, indicative of protein and lipid oxidative damage, were higher in endometriosis group (21%, *p* = 0.016 and 46%, *p* = 0.009, respectively). However, these biomarkers were not different in the blood plasma of both groups, and only protein dityrosine, was increased in the plasma of endometriosis group (31%, *p* = 0.04). The peritoneal fluid hemoglobin content was not higher in the endometriosis group, nor related to carbonyls and lipohydroperoxides. Additionally, the peritoneal fluid oxidative biomarkers were not correlated with the blood plasma ones, and only malondialdehyde, and ischemia-modified albumin were almost two times higher in peritoneal fluid.

**Discussion:** Our results show a peritoneal and systemic oxidative stress biomarkers increase in endometriosis, but not related to each other, and do not support the hypothesis of an increase in hemoglobin-iron supply towards the peritoneal cavity that causes oxidative damage.

## Introduction

Endometriosis is a gynecological disorder characterized by the growth of endometrial glands and stromal cells outside the uterus, generally in the peritoneal cavity, but it can also occur in other places such as the liver, kidney, pleural cavity or bladder [1]. The prevalence of this disease ranges from 6% to 15% during reproductive age; but in infertile women has been reported a range between 20% and 50%, likely because infertile women are more prone to undergo laparoscopy, the definitive test for endometriosis diagnosis [2]. In addition to pelvic pain and dysmenorrhea, endometriosis affects the reproductive process at different levels (ovarian, tubal function and uterine receptivity) by means of diverse suggested mechanisms [3]. One mechanism is likely oxidative stress; that is, the imbalance between the production of reactive oxygen species (ROS) and antioxidant defense, which can result from diminished levels of antioxidants and/or an increased production of ROS, causing oxidative stress-dependent damage to biomolecules and immune inflammatory response [4,5]. This condition is likely to exist in the peritoneal cavity because of the retrograde menstruation, an endometrial cells and tissue reflux through fallopian tubes into the peritoneal cavity causing leukocytosis and excessive iron liberated by erythrocyte lysis [6]. This metal can act as a catalyst in the Fenton reaction generating the hydroxyl radical, one of the most powerful ROS. In addition, there is evidence that oxidative stress is not restricted to the peritoneal cavity but is a more peripheral condition [5]. It is possible that a systemic oxidative stress condition linked to endometriosis may be due to the permeability properties of the peritoneum [7]; that is, the inflammatory condition that exists in the peritoneal cavity of patients with endometriosis produces ROS that modify diverse biomolecules, which could be transported to the systemic circulation through the peritoneal membrane. Moreover, there is hard evidence of a large flux of endometrial and leukocyte cells and iron towards the peritoneal cavity by means of retrograde menstruation [8], and that the anatomical distribution of endometriotic lesions shows a linkage with the peritoneal fluid flow [9]. However it is not clear if the retrograde menstruation is increased in patients with endometriosis. To test these possibilities, we compared the levels of oxidative stress biomarkers in peritoneal fluid versus peripheral blood plasma of the two patients groups. Additionally, the concentrations of hemoglobin and erythrocytes, and the volume of peritoneal fluid of endometriosis and no endometriosis patients were compared. We also compared the levels of total proteins and human serum albumin concentrations between blood plasma and peritoneal fluid to determine possible changes in peritoneum permeability associated to endometriosis.

## Materials and methods

This transversal and observational study was carried out at the Instituto Nacional de Perinatología ‘Isidro Espinosa de los Reyes’ (INPerIER) in Mexico City, México. The study was performed according to the principles of The Declaration of Helsinki and was approved by INPerIER's Research and Ethic Commissions and registered with the number 3210-21009-08-15, 29 September 2015.

### Patients

Patients who were 18–35 years old from the Infertility Clinic and other patients above 35 years were recruited from the Gynecology Department, from the INPerIER in Mexico City, and who gave written informed consent were included in the study. We included patients with suspected endometriosis due to clinical symptoms (chronic pelvic pain, dysmenorrhea, dyspareunia and infertility), and scheduled for diagnostic or surgical laparoscopy.

Endometriosis was diagnosed via laparoscopy, and severity was classified during surgery according to the American Society Criteria for Reproductive Medicine [10]. Infertility was also determined in accordance with the American Society for Reproductive Medicine Criteria. Patients were excluded from the study in the following cases: autoimmune, endocrine or metabolic diseases, tobacco or alcohol consumption, the presence of pelvic inflammatory disease, or the use of anti-inflammatory or multivitamin supplements.

#### Collection of peritoneal fluid and blood samples

Peritoneal fluids were collected by aspiration the pouch of Douglas from the cul-de-sac during laparoscopy. Only peritoneal fluids without dilution and without hemorrhage due to the insertion of trocars were retrieved to avoid dilution and contamination with peripheral blood. The peritoneal fluids were centrifuged at 2500 rpm for 10 min at 4°C, and peritoneal fluid supernatants were collected and stored in aliquots at −70°C until their analysis. Whole blood 5 mL samples were obtained from antecubital venipuncture in anticoagulated tubes with 2 mM EDTA (BD Vacutainer, USA) before laparoscopy, and the samples were centrifuged at 2500 rpm for 10 min to obtain the plasma within 1 h of collection.

#### Biomarkers of oxidative stress

To determine the oxidative stress status of the patients, carbonyls, dityrosine and ischemia-modified albumin (IMA), indicative of oxidative-modified proteins; and malondialdehyde and lipohydroperoxides, indicative of lipoperoxidation, were measured.

Protein carbonylation was determined by the dinitrophenylhydrazine method [11]. Briefly, 0.05 mL of peritoneal fluid or plasma was mixed with 0.5 mL 10 mM dinitropenylhydrazine (DNPH) in 2.5 M HCl (or 2.5 M HCl alone for the blank). Samples were left for 1 h at ambient temperature, and then 0.5 mL 20% trichloroacetic was added and centrifugated at 3000 rpm, 5 min, 4 C. The resultant pellet was rinsed twice by centrifugation with 1 mL 5% TCA. The pellet was washed by centrifugation with 2 mL ethanol/ethylacetate (1:1) and solubilized in 0.5 mL of 6 M guanidine in 20 mM potassium phosphate, pH 2.3. The carbonyl concentration was determined using the extinction molar coefficient *ε* = 22 mM^−1^ cm^−1^ and expressed as n mol/mg protein. Dityrosine was measured by fluorescence according to [12]: 50 µL of plasma or peritoneal fluid were mixed with 1950 µL of 6 M urea–0.1 M sodium bicarbonate pH 9.8, and after 30 min at ambient temperature, the fluorescence was measured in a fluorometer (Turner Designs TD-700) previously adjusted with 1 µM quinine sulfate, with E_x_ 320–E_m_ 405 filters. The dityrosine concentration was calculated with a standard curve realized with dityrosine synthesized according to [12], and expressed as n mol/mg protein.

IMA was determined with minor modifications of the original method of Bar-Or D [13]: 50 µL plasma or peritoneal fluid were mixed with 25 µL cobalt chloride (CoCl_2_-6 H_2_O) 0.1% in Hepes 0.1 M pH 7.5, and incubated for 10 min at room temperature; then 25 µL dithiothreitol 1.5 mg/mL in Hepes 0.1 M pH 7.5 (or 25 µL of Hepes alone for the blank) were added and mixed. After 4 min at room temperature, 0.5 mL NaCl 0.9% were added, and the absorbance at 470 nm were measured. These modifications were realized to regulate the assays pH at 7.5 values, as suggested by Lee et al. [14]. IMA values were expressed as absorbance units (AU)/mg of human serum albumin. Human serum albumin (HSA) was measured by the Bromocresol Green method [15]. Lipohydroperoxides (LHP) and malondialdehyde (MDA) were measured according to the methods mentioned in [16,17], respectively. The proteins were determined using the method of Lowry [18], and dry weight using the colorimetric method of Bernal [19].

#### Statistics

The data are expressed as the means ± SD. A one-way analysis of variance and the Bonferroni multiple comparison test, as well as linear regression and Pearson correlation were used for statistical analysis. Differences were considered significant when *p* < 0.05. The data were analyzed using the Statistical Package for Social Sciences (version 10.0 for Windows; SPSS, Inc., Chicago, IL).

## Results

Table 1 shows the demographic and clinic characteristics of the studied patients. A total of 51 patients with (*n* = 28) or without (control group, *n* = 23) endometriosis were included. In the no endometriosis group, 13 patients were infertile and 18 in the group with endometriosis. Minimal (46.4%) and severe (35.7%) were the most frequently stages of endometriosis. Other characteristics are shown, but there were no significant demographic differences between groups.

**Table 1.** Demographic and clinical characteristics.

	No endometriosis (*n* = 23)	Endometriosis (*n* = 28)	*p* value
Age (years)	31.9±7.2	34.4±6.3	0.220
BMI (kg/m^2^)	27.2±3.9	25.6±4.9	0.217
Infertility (%)	13 (56.5)	18 (64.2)	0.636
Primary infertility (%)	9 (69.2)	10 (55.5)	
Secondary infertility (%)	4 (30.7)	8 (44.4)	
Infertility time (years)	2.1±2.9	3±3.8	0.400
Oral contraceptive (%)	13 (56.5)	14 (50)	0.642
Dysmenorrhea (%)	11 (47.8)	20 (71.4)	0.121
AFS endometriosis staging			
Minimal (%)		13 (46.4)	
Mild (%)		2 (7.1)	
Moderate (%)		3 (10.7)	
Severe (%)		10 (35.7)	

Data are presented as mean±SD.

To determine if the studied patients were in a systemic oxidative stress condition linked to the endometriosis occurrence, some oxidative stress biomarkers were measured in the plasma from peripheral blood (P) and in the peritoneal fluid (PF). Table 2 shows the values of the oxidative stress biomarkers in the peritoneal fluid. Only carbonyls and the lipohydroperoxides of PF of patients with endometriosis were significantly higher than the without endometriosis group. Other parameters of peritoneal fluid were analyzed between groups, to determine if the endometriosis occurrence has any influence on these variables. Total protein and HSA concentrations in peritoneal fluid were not different between the two groups, nor hemoglobin and volume. Although the median value of erythrocytes was greater in the peritoneal fluid of endometriosis group, it was not statistically significant because of the great dispersion of the data (Table 2). In order to determine the possible effect of the use of oral contraceptives, obesity and infertility on biomarkers of oxidative damage, we realized a multivariate analysis. We found a significant decrease in peritoneal fluid carbonyls in the group of patients who used oral contraceptives (4.8 ± 1.4 vs 6.3 ± 2.5 nmol/mg protein, *p* = 0.015). We did not find significant differences related to BMI, but peritoneal fluid carbonyls increased significantly in infertile patients (6.0 ± 2.2 vs 4.8 ± 0.9 nmol/ mg protein, *p* = 0.028).

**Table 2.** Oxidative stress biomarkers and other characteristics of peritoneal fluid.

	No endometriosis (*n* = 23)	Endometriosis (*n* = 28)	Statistical significance
Protein (mg/mL)	56.84 (±15.08)	63.76 (±19.02)	NS
HSA (mg/mL)	29.37 (±6.279)	30.26 (±5.020)	NS
Carbonyls (nmol/mg protein)	4.66 (±1.15)	5.64 (±1.24)	0.016
MDA (nmol/mg dry weight)	0.1905 (±0.0558)	0.2211 (±0.0609)	NS
LHP (pmol/mg dry weight)	3.300 (±1.157)	4.848 (±2.068)	0.009
DT (nmol/mg protein)	0.5780 (±0.2609)	0.6074 (±0.3324)	NS
SH (nmol/mg protein)	4.690 (±1.281)	5.127 (±1.998)	NS
IMA (AU/mg HSA)	0.2654 (± 0.1040)	0.2305 (±0.0471)	NS
Hb (mg/mL)	0.8366 (±0.4934)	1.941 (±2.098)	NS
Erythrocytes (10^6^/µL)	0.1142 (±0.2137)	0.2362 (±0.3217)	NS
Volume (mL)	5.722 (±4.059)	5.532 (±4.018)	NS

Data are presented as means ± SD. NS, Not significant difference; HSA, Human Serum Albumin; MDA, Malondialdehyde; LHP, Lipohydroperoxides; DT, Dityrosines; SH, Sulfhydryls; IMA, Ischemia-Modified Albumin; Hb, Hemoglobin.

Table 3 shows the blood plasma parameters measured. Only dityrosine was slightly elevated in endometriosis group. In order to determine if endometriosis modifies the oxidative stress biomarkers distribution between blood plasma and peritoneal fluid, in Table 4 we show the ratios of blood plasma/peritoneal fluid for proteins and for oxidative stress biomarkers. The total protein and HSA concentrations were significantly higher (1.46–1.31 times for total proteins and 1.4–1.37 times for HSA) in blood plasma than in peritoneal fluid, as like reported by Kelton group [7]; but no differences within the endometriosis and no endometriosis groups were found. Likewise, the oxidative stress biomarkers were not differently distributed between blood plasma and peritoneal fluid in the two groups; however, carbonyls, LHP and DT were higher in blood plasma than in peritoneal fluid: 1.4 and 1.7 for carbonyls, 2.1 and 1.7 for LHP, 2.1 and 2.7 for DT, in no endometriosis and endometriosis groups, respectively. On the contrary, MDA and IMA were more distributed towards the peritoneal fluid, with ratios of blood plasma/peritoneal fluid of 0.57 and 0.48 for MDA, and 0.56 and 0.66 for IMA, in control and endometriosis groups, respectively (Table 4). Additionally, to determine if the oxidative stress biomarkers of peritoneal fluid and blood plasma were quantitatively related, Pearson correlations between these biomarkers were done, and no significant correlation was found.

**Table 3.** Blood plasma oxidative stress markers and other characteristics.

	No endometriosis (*n* = 23)	Endometriosis (*n* = 28)	Statistical significance
Protein (mg/mL)	83.18 (±10.83)	83.64 (±13.80)	NS
HSA (mg/mL)	41.28 (±5.197)	41.60 (±4.533)	NS
Carbonyls (nmol/mg protein)	8.20 (±2.50)	7.88 (±3.06)	NS
MDA (nmol/mg dry weight)	0.1099 (±0.032)	0.1073 (±0.037)	NS
LHP (pmol/mg dry weight)	7.045 (±2.703	8.525 (±3.264)	NS
DT (nmol/mg protein)	1.258 (±0.267)	1.651 (±0.739)	0.04
SH (nmol/mg protein)	5.284 (±0.5797)	5.408 (±1.077)	NS
IMA (AU/mg HSA)	0.1490 (±0.0219)	0.1533 (±0.022)	NS

Abbreviations are the same as Table 2.

**Table 4.** Protein and oxidative stress biomarkers distribution.

Characteristic	No endometriosis	Endometriosis
Total protein	1.46	1.31
HSA	1.40	1.37
Carbonyls	1.76	1.40
MDA	0.57	0.48
LHP	2.13	1.76
DT	2.17	2.72
SH	1.13	1.05
IMA	0.56	0.66

Distribution is expressed as the ratio Plasma/Peritoneal Fluid.

Abbreviations are the same as in Table 2.

## Discussion

Oxidative stress is a condition that is caused by an imbalance between oxidants (ROS) and antioxidants in favor of the former, which leads to potential damage [4], and this condition has been documented in endometriosis [4,5]. In this study, we hypothesized that the inflammatory status of peritoneal cavity associated to endometriosis may oxidize diverse biomolecules, which can cross to the peripheral blood, and contribute to the increase of peripheral oxidative stress biomarkers. If so, we expect these oxidative stress biomarkers to be higher in the peritoneal fluid than in the blood plasma of the patients with endometriosis. We measured six oxidative stress biomarkers and we found that only carbonyls and lipohydroperoxides (biomarkers of ROS-induced protein and lipid damage, respectively) had higher levels in the peritoneal fluid of endometriosis group. However, these biomarkers were not higher in the blood plasma of endometriosis patients, and only the dityrosines was slightly increased in the plasma of this group. In addition, except for MDA and IMA, the distribution of these oxidative stress biomarkers between blood plasma and peritoneal fluid (expressed as the ratio of plasma/peritoneal fluid, Table 4) was higher in the plasma, not in the peritoneal fluid. Moreover, the Pearson correlation coefficients of the oxidative stress biomarkers of peritoneal fluid and blood plasma did not show significant differences in both groups studied. Taking together, these facts do not support the hypothesis of peritoneal origin of the peripheral increase of these oxidative stress biomarkers. Interestingly, MDA and IMA were around 1.5–2 times higher in the peritoneal fluid than in the blood plasma, although without differences between both patient groups. Why MDA and IMA are increased in peritoneal fluid, independently of endometriosis presence? It is unknown. Possibly, the free iron-rich and hypoxic ambient prevalent in the peritoneal cavity contribute to MDA and IMA increases; however, in vitro addition and incubation of hemoglobin or erythrocytes lysate with blood plasma or peritoneal fluid, do not increase MDA nor IMA detection (unpublished data). Roy et al. [20] have reported that hydroxyl radical can induce IMA formation in vitro, but in vivo this fact has not been proved, even though activated immune cells at the peritoneal cavity could be a source of this kind of oxygen radical.

The etiology and pathogenesis of endometriosis remains unclear. Retrograde menstruation is the main and the oldest theory proposed, but other theories have been suggested, like celomic metaplasia, lymphovascular metastasis, embryonic rest theory and altered immune response [21]. Retrograde menstruation is the regurgitation of endometrial cells, blood components and cellular debris through the fallopian tubes into the peritoneal cavity during menstruation. Due to approximately 75–90% of women experience this physiological process [22], it appears to be a very common phenomenon and this theory cannot explain why only certain women develop endometriosis. Then, it is clear that there must be other unknown factors which may contribute to the ectopic implantation of endometrial cells and their subsequent development. A high and frequent retrograde menstruation could be a factor for the development of endometriosis; however, in our study the total protein and HSA concentrations in peritoneal fluid were not different between the two studied groups, nor hemoglobin, and peritoneal fluid volume. These results do not support the hypothesis of a more large retrograde menstruation related to endometriosis.

A possible confusion factor for this study may be the day of the menstrual cycle phase in which the samples was taken. This fact is important because it is well documented that estrogens have a protective effect against oxidative damage [23]. However, other study reported no differences on peritoneal oxidative stress biomarkers levels related to menstrual cycle phases [24]. We analyzed the possible effect of the use of oral contraceptives on biomarkers of oxidative damage. We found a decrease of peritoneal fluid carbonyls in the group of patients who used oral contraceptives, suggesting a protective effect of estrogen against carbonyls formation. We also analyzed the possibility of whether other variables such as BMI or infertility could be related to the biomarkers studied. We did not find significant differences related to BMI, but peritoneal fluid carbonyls increased significantly in infertile patients.

In addition, another limitation of the study is the fact that, according to the biomarkers studied, it is not possible to determine which reactive oxygen species are involved, nor to establish their source. It is possible that the inflammatory condition that prevails in the peritoneal cavity of patients induces the recruitment of leukocytes and the release of proinflammatory cytokines that activate enzymes such as NADPH oxidase and myeloperoxidase. These enzymes produce superoxide anion/hydrogen peroxide, and hypochlorite, respectively, and its possible contribution in the increase of biomarkers of oxidative damage in endometriosis will have to be established.
